# Soluble immune checkpoints are elevated in patients with primary biliary cholangitis

**DOI:** 10.1186/s40001-023-01419-6

**Published:** 2023-11-02

**Authors:** Xiuzhu Gao, Xiaomei Wang, Yazhe Guan, Liquan Wang, Yanhang Gao, Junqi Niu

**Affiliations:** 1 Department of Hepatology, First Hospital of Jilin University, Jilin University, No 71 Xinmin Street, Changchun, 130021 Jilin China; 2 Jilin Province Occupational Disease Prevention and Treatment Hospital, 2351 Mingxi Road, Changchun, 130052 China

**Keywords:** Primary biliary cholangitis, CD134, LAG-3, PD-1, PD-L1, TIM-3

## Abstract

**Background:**

Primary biliary cholangitis (PBC) is a chronically progressive liver disease mediated by an autoimmune response. The aetiology and pathogenesis of PBC are not fully understood and may be related to immune disorders caused by genetic factors and their interaction with environmental factors. Immune checkpoints play an important role in preventing the occurrence of autoimmunity. However, the level of immune checkpoints in PBC has not been reported. Here, we aimed to identify the serum levels of soluble checkpoints in patients with PBC.

**Methods:**

Soluble checkpoint levels were evaluated using enzyme-linked immunosorbent assay in 60 patients with PBC and 20 healthy controls (HCs). The expression of immune checkpoints was compared in liver biopsy tissue samples using immunohistochemistry. Receiver operating characteristic (ROC) curves and area under the curve (AUCs) were used to determine the diagnostic performance of soluble checkpoints and laboratory indexes between patients with PBC and HCs and patients with mild and advanced PBC. A logistic regression was performed for advanced PBC.

**Results:**

sCD134, sLAG-3, sPD-1, sPD-L1, and sTIM-3 levels were significantly increased in patients with PBC compared with those in healthy controls. Additionally, the levels of sCD134, sPD-1, sPD-L1, and sTIM-3 were positively associated with disease progression. Moreover, soluble checkpoints were correlated with immunoglobulin and liver functions. ROC analyses between patients with PBC and HCs showed that the AUCs of sOX40, sPD-1, and sPD-L1 were 0.967, 0.922, and 0.971, respectively. The optimal cut-off values of sOX40, sPD-1, and sPD-L1 for PBC diagnosis were 89.15, 213.4, and 68, respectively. ROC analyses between mild and advanced patients with PBC revealed that the AUCs of sOX40 and sTIM-3 were 0.767 and 0.765, respectively. The optimal cut-off values for predicting PBC stage ≥ III were 199.45 and 361.5, respectively. In univariate analysis, age, ALB, and sOX40 were associated with advanced PBC. Further, the expression of CD134 and TIM-3 was upregulated in the liver of patients with PBC.

**Conclusions:**

Our study results indicate that the serum titer of soluble checkpoints is increased in Chinese patients with PBC.

**Supplementary Information:**

The online version contains supplementary material available at 10.1186/s40001-023-01419-6.

## Background

Primary biliary cholangitis (PBC) is a chronically progressive, cholestatic liver disease characterised by non-suppurative inflammation mediated by an autoimmune response [1]. It mostly occurs in middle-aged women [2]. The onset of PBC is insidious and slow, and the prognosis varies greatly. Additionally, serum alkaline phosphatase (ALP) and gamma‐glutamyl transferase (γGT) increase to varying degrees. Anti-mitochondrial antibody (AMA) is the autoantibody with the highest specificity of PBC [3]. However, some patients with PBC test negative for AMA.

Immune checkpoints comprise a suite of molecules expressed on immune cells that can regulate immune responses. They play an important role in preventing the occurrence of autoimmunity [4]. The abnormal expression of immune checkpoints can lead to the development of tumours or autoimmune diseases. Two kinds of checkpoint molecules are related to immune regulation: stimulatory checkpoint molecules and inhibitory checkpoint molecules. Checkpoint molecules with negative immunoregulatory effects include CTLA-4, TIM-3, PD-1, PD-L1, LAG-3, and VISTA. Additionally, checkpoint molecules belonging to the tumour necrosis factor (TNF) superfamily, such as GITR and OX40, have been introduced as stimulatory factors (PMID: 33009134. To date, most studies on PBC and immune checkpoints have focused on CTLA-4 [5, 6], and there are few studies on other immune checkpoints.

This study aims to compare the levels of soluble immune checkpoints in the serum of patients with PBC and healthy controls. Additionally, we analysed and compared their correlation with the severity of PBC and laboratory indicators. ROC analyses were performed to evaluate the diagnostic accuracy of serum OX40, PD-1, PD-L1, and TIM-3 for PBC staging determination. Finally, the expression of immune checkpoints in liver biopsy tissue samples was compared between patients with PBC and healthy controls to explore the predictive role of immune checkpoints in the progression of PBC.

## Methods

### Patients with PBC and healthy controls

This study involved 80 individuals (60 patients with PBC and 20 HCs) recruited from The First Hospital of Jilin University between 2014 and 2016. The patients were diagnosed with PBC based on the American Association for the Study of Liver Diseases Practice Guideline, and all the subjects were untreated. The experimental protocol was approved by the Ethics Committee of the First Hospital of Jilin University (Changchun, China) under the project approval number 2014–424. All participants signed an informed consent form. Our protocol was performed in accordance with the guidelines and regulations of the Declaration of Helsinki. According to the Ludwig staging system of histopathology, PBC progression was divided into four stages. However, some biopsies were classified between 1 and 2, 2 and 3 and 3–4; hence, the stage was defined as 1.5, 2.5 and 3.5, respectively. Blood samples were collected from each individual, centrifuged at 4000 ×*g* for 10 min to obtain serum and stored at – 80 °C until use. The baseline characteristics of patients with PBC and healthy control are described in Table 1. More details are available in Additional file 1: Table S1.

**Table 1:** Patients’ clinical characteristics and laboratory index

	PBC (N = 60)	HC (N = 20)	*P* value
Age,mean(SE)	54.4 ± 1.2	53.4 ± 1.6	0.197
Sex			
Male (%)	8 (13.3%)	5 (25%)	0.293
Female (%)	52 (86.7)	15 (75%)	
Histological stage			
1	11	–	–
2	24	–	–
3	8	–	–
4	11	–	–
Unclear	6	–	–
ALT(U/L, median(range))	55.5 (13–893)	22.6 (9.9–109)	< 0.0001
γGT(U/L, median(range))	210.5 (20–1218)	19.6 (5–80)	< 0.0001
ALP(U/L, median(range))	186.1 (67–1081)	67.2 (42–128)	< 0.0001
AST(U/L, median(range))	76 (21–730)	24 (17.1–66)	< 0.0001
Glb(g/L, median(range))	35 (23–257)	29.3 (25.3–42)	< 0.0001
TB(μmol/L, median(range))	27.6 (5.5–282.8)	14.05 (7.7–32.6)	< 0.0001
DBIL(μmol/L, median(range))	11.2 (2–168.3)	2.35 (1.4–4.9)	< 0.0001
IBIL(μmol/L, median(range))	13 (3–114.5)	11.85 (6.3–27.7)	< 0.0001
TBA(μmol/L, median(range))	34 (0.4–268.6)	2.85 (1.7–14.8)	< 0.0001
CHE(U/L, median(range))	5945 (374–12,580)	8495 (6729–12,532)	0.0114
IgA(g/L, median(range))	3.2 (1.4–9.6)	NA	NA
IgG(g/L, median(range))	15.9 (10.5–56.1)	NA	NA
IgM(g/L, median(range))	2.7 (0.8–8.1)	NA	NA

### Enzyme-linked immunosorbent assay (ELISA)

Concentrations of soluble CD134, LAG-3, PD-1, PD-L1, and TIM-3 in plasma were determined using ELISA (Abcam, US) following the established protocol. The calculated minimal detectable doses were 26 pg/mL, 0.045 pg/mL, 9.6 pg/mL, 2.91 pg/mL and 31.2 pg/mL, respectively. The concentrations of soluble checkpoints in individual samples were calculated according to the standard curve constructed using the recombinant checkpoint provided.

### Liver biopsy

To ensure the quality of histological results and minimise differences in histopathological evaluation, two pathologists were blinded to clinical data according to the standard operation procedure. Single-blind radiographs by more than two liver pathologists are recommended.

### Immunohistochemistry

Liver biopsy samples were collected from patients with PBC and their counterpart negative controls with hepatic hemangioma without any other liver disease. Intrahepatic CD134, LAG-3, PD-1, PD-L1, and TIM-3 were visualised through immunohistochemical staining of the tissues embedded in paraffin using rabbit anti-human CD134, LAG-3, PD-1, PD-L1, TIM-3 antibodies (Abcam) and Envision + System, HRP (diaminobenzidine) (DAKO). Checkpoint quantification for each pathological section was determined by two pathologists blinded to clinical and molecular data using a modified H-Score to determine the overall percentage of protein positivity across the entire stained sample. Pi represents the percentage of pixel area with positive signals; I stands for positive level. The H-score ranges from 0 to 300, and the greater the value, the stronger the comprehensive positive intensity [7].

### Statistical analysis

For statistical analyses, differences in continuous variables between two groups were evaluated using the Mann–Whitney U test or a two-sided Student’s t-test, depending on the data characteristics. Dichotomous variables were compared using the χ2 test. Spearman’s rank correlation test assessed the correlations between soluble immune checkpoints and biochemical indexes. ROC curve and AUCs were used to determine the diagnostic performance of soluble checkpoints and laboratory indexes between patients with PBC and HCs and those with mild and advanced PBC, respectively. The optimal cut-offs were determined based on the sensitivity and specificity. For logistic regression analysis of advanced PBC, all variables found to be significant (*P* ≤ 0.05) through univariate analysis were considered for inclusion in multivariable analysis. A backward stepwise logistic regression was performed, and statistically significant factors (*P* ≤ 0.05) in multivariable analysis remained in the final model. Odds ratio (OR) with 95% Confidence Interval (CI) were presented to demonstrate the strength and direction of these associations. The statistical software GraphPad Prism 7.0 (GraphPad Software, La Jolla, CA, USA) was used for graph creation. A p-value < 0.05 was considered statistically significant. p values are indicated as * < 0.05; ** < 0.01; and *** < 0.001.

## Results

### Primary characteristics of participants

Eighty participants were enrolled in this study. The cohort comprised 60 patients with PBC and 20 healthy controls (HCs) with average ages of 54.4 and 53.4 years, respectively. The PBC group consisted of 8 males and 52 females, while the HC group included 5 males and 15 females (*P* = 0.29). All participants were of Chinese descent. Patients diagnosed with PBC underwent clinical evaluations, while individuals with common liver and other diseases were excluded from the HC group (refer to Table 1 for further details). Additional information can be found in Additional file 1: Table S1.

### Elevated levels of soluble immune checkpoint-related proteins in patients with PBC

**Fig. 1:**
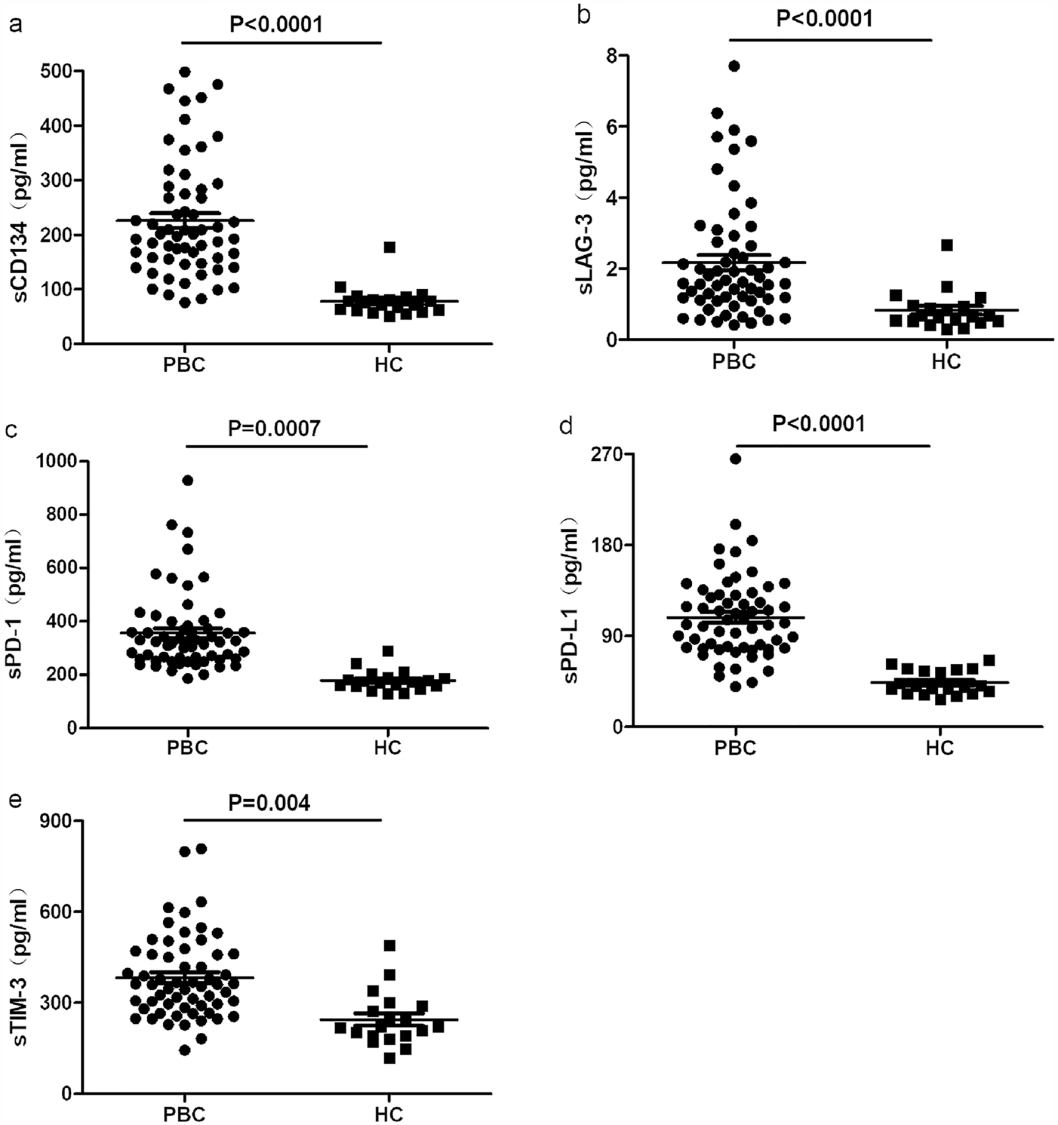
The levels of soluble immune checkpoints, including CD134 (**a**), LAG-3 (**b**), PD-1 (**c**), PD-L1 (**d**), and TIM-3 (**e**), observed in patients diagnosed with PBC and a control group of healthy individuals

**Fig. 2:**
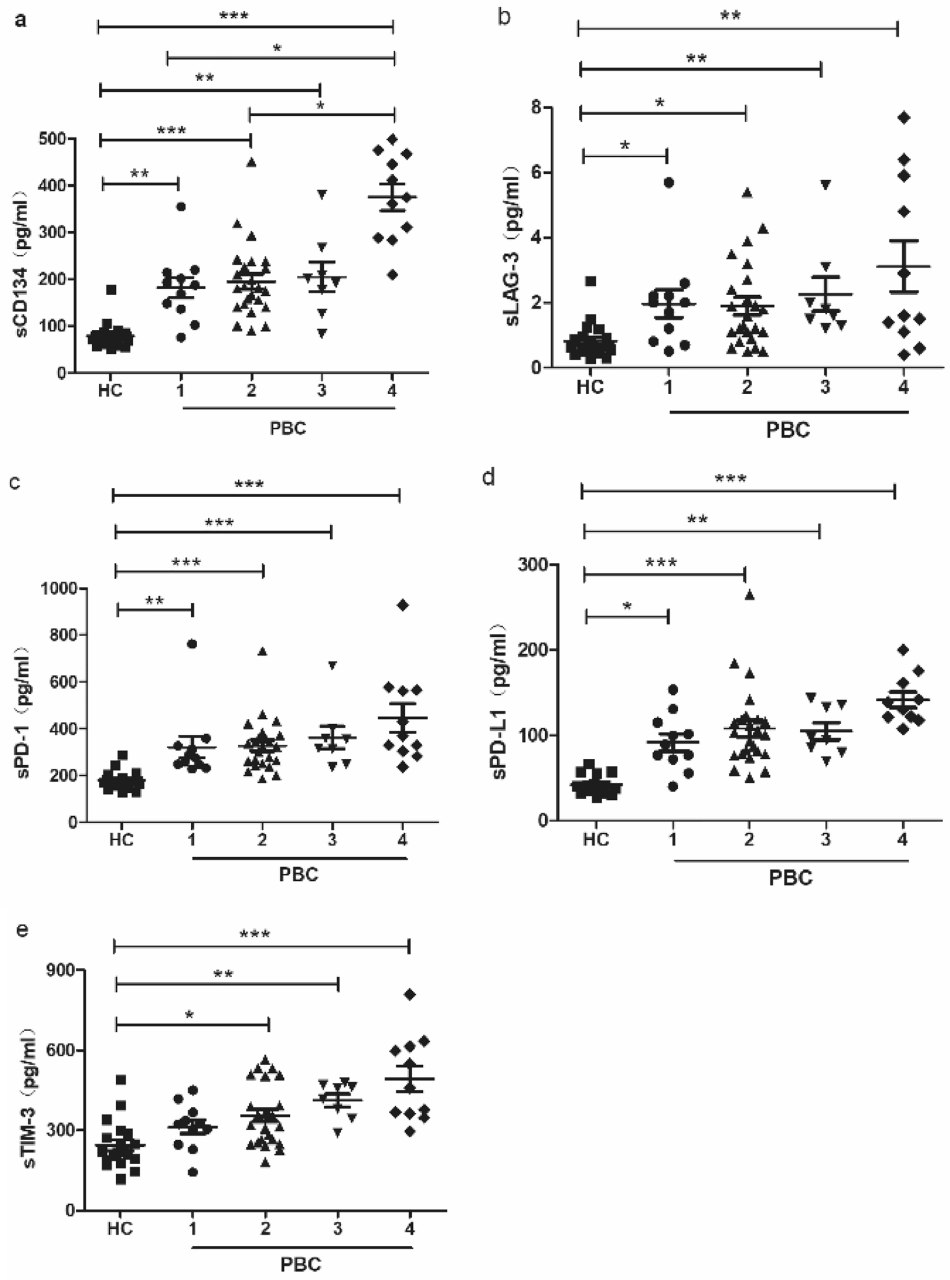
The levels of CD134 (**a**), LAG-3 (**b**), PD-1 (**c**), PD-L1 (**d**), and TIM-3 (**e**) in patients with PBC at different stages

In the initial analysis, we conducted a comparative assessment of soluble immune checkpoint protein concentrations in patients with PBC and healthy individuals. As illustrated in Fig. 1, we observed significantly higher levels (P < 0.05) of soluble CD134 (a), LAG-3 (b), PD-1 (c), PD-L1 (d), and TIM-3 (e) in patients with PBC compared to those in the HC group. Additionally, participants were categorised into four distinct disease stage groups. As depicted in Fig. 2, CD134 exhibited significantly elevated levels in individuals diagnosed with stage IV PBC compared to those in other stages.

### Correlation between soluble immune checkpoint levels and immunoglobulin (Ig) and liver function

We conducted correlation analysis to assess the relationship between sCD134 levels and various parameters related to immunoglobulin levels and liver function in Table 2. sCD134 levels displayed a positive correlation with titers of IgA and IgG, both of which are associated with autoimmune diseases. Elevated levels of IgA can be indicative of liver inflammation. Furthermore, serum levels of total bilirubin (TB), direct bilirubin (DBIL), and indirect bilirubin (IBIL), which are known to increase in patients with hepatic diseases, were also positively correlated with sCD134 levels. Interestingly, sCD134 was negatively correlated with serum cholinesterase (CHE) levels, an indicator of liver function.

**Table 2:** Correlation between soluble immune checkpoint levels and immunoglobulin (Ig) and liver function

	CD134		PD-1		PD-L1		LAG-3		TIM-3	
	*r*	*P*	*r*	*P*	*r*	*P*	*r*	*P*	*r*	*P*
ALP	− 0.13	0.34	0.38	0.01	0.38	0.01	− 0.24	0.08	0.19	0.18
IgA	0.34	0.03	0.15	0.40	0.16	0.31	0.31	0.06	− 0.03	0.85
IgG	0.34	0.04	0.48	0.00	0.48	0.00	0.52	0.00	− 0.07	0.68
IgM	− 0.097	0.56	0.14	0.42	− 0.027	0.87	0.27	0.12	0.18	0.29
AST	0.18	0.19	0.39	0.00	0.39	0.00	0.36	0.01	0.19	0.15
TB	0.33	0.01	0.26	0.06	0.23	0.08	0.24	0.08	0.56	< 0.0001
TBA	0.23	0.09	0.45	0.00	0.45	0.00	0.41	0.00	0.01	0.96
CHE	− 0.5	< 0.0001	− 0.31	0.02	− 0.31	0.02	− 0.37	0.01	− 0.37	0.00

Our data revealed several correlations between sLAG-3 levels and parameters related to immunoglobulin levels and liver function. Firstly, there is a positive correlation between sLAG-3 levels and IgG levels. Additionally, sLAG-3 levels were significantly positively associated with aspartate aminotransferase (AST), which is commonly used as a biochemical indicator of inflammatory fluctuations in hepatitis. Moreover, serum total bile acid (TBA) and TBI levels were positively correlated with sLAG-3 levels. Lastly, a negative correlation was observed between sLAG-3 levels and serum cholinesterase (CHE) levels, an indicator of liver function.

Our correlation analysis revealed several associations between sPD-1 and sPD-L1 levels and parameters related to immunoglobulin levels and liver function. Firstly, sPD-1 levels displayed positive correlations with levels of IgA, AST, DBIL, IBIL, TBA, and alkaline phosphatase (ALP). Additionally, both sPD-1 and sPD-L1 levels exhibited a negative correlation with CHE levels, an indicator of liver function.

Our correlation analysis reveals several associations between sTIM-3 levels and parameters related to liver function. Specifically, sTIM-3 levels displayed positive correlations with TB, DBI, IBIL, and globulin (GLB). Moreover, a negative correlation was observed between sTIM-3 levels and CHE levels, an indicator of liver function.

### Correlation among soluble immune checkpoints

**Fig. 3:**
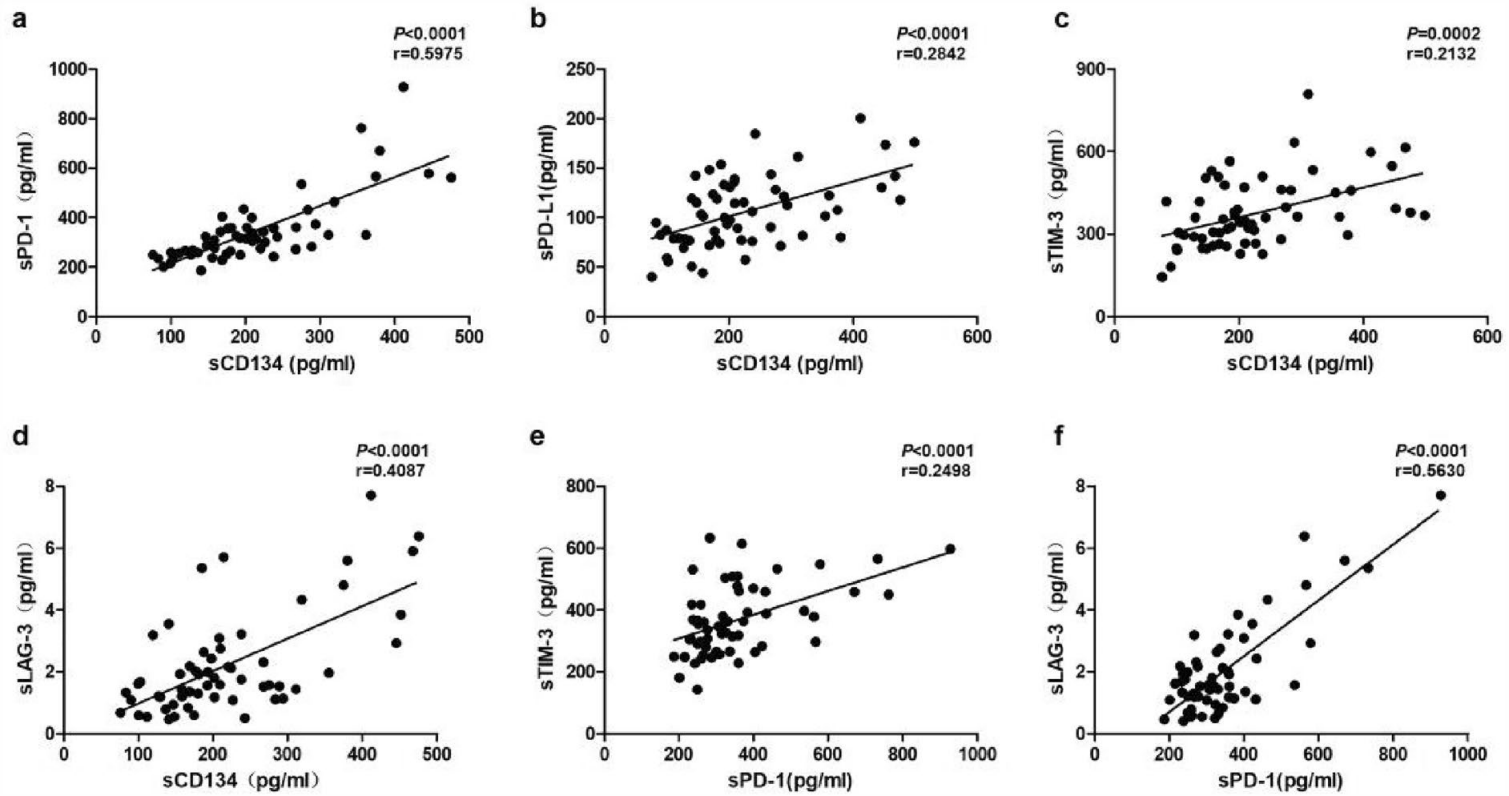
Correlations between sOX40 levels and sPD-1(**a**), sPD-L1 (**b**), sTIM-3 (**c**) and sLAG-3 (**d**); and between sPD-1 and sTIM-3 (**e**) and sLAG-3 (**f**)

Our correlation analysis revealed several associations among the soluble immune checkpoints. Firstly, sOX40 levels displayed positive associations with sPD-1, sPD-L1, sTIM-3, and sLAG-3 (Fig. 3a, b, c, d). Additionally, sPD-1 levels were positively associated with sTIM-3 and sLAG-3 (Fig. 3e, f).

### The diagnostic efficiency of serum soluble checkpoints and laboratory indexes for PBC and predicting its stage evaluated using ROC analyses

**Fig. 4:**
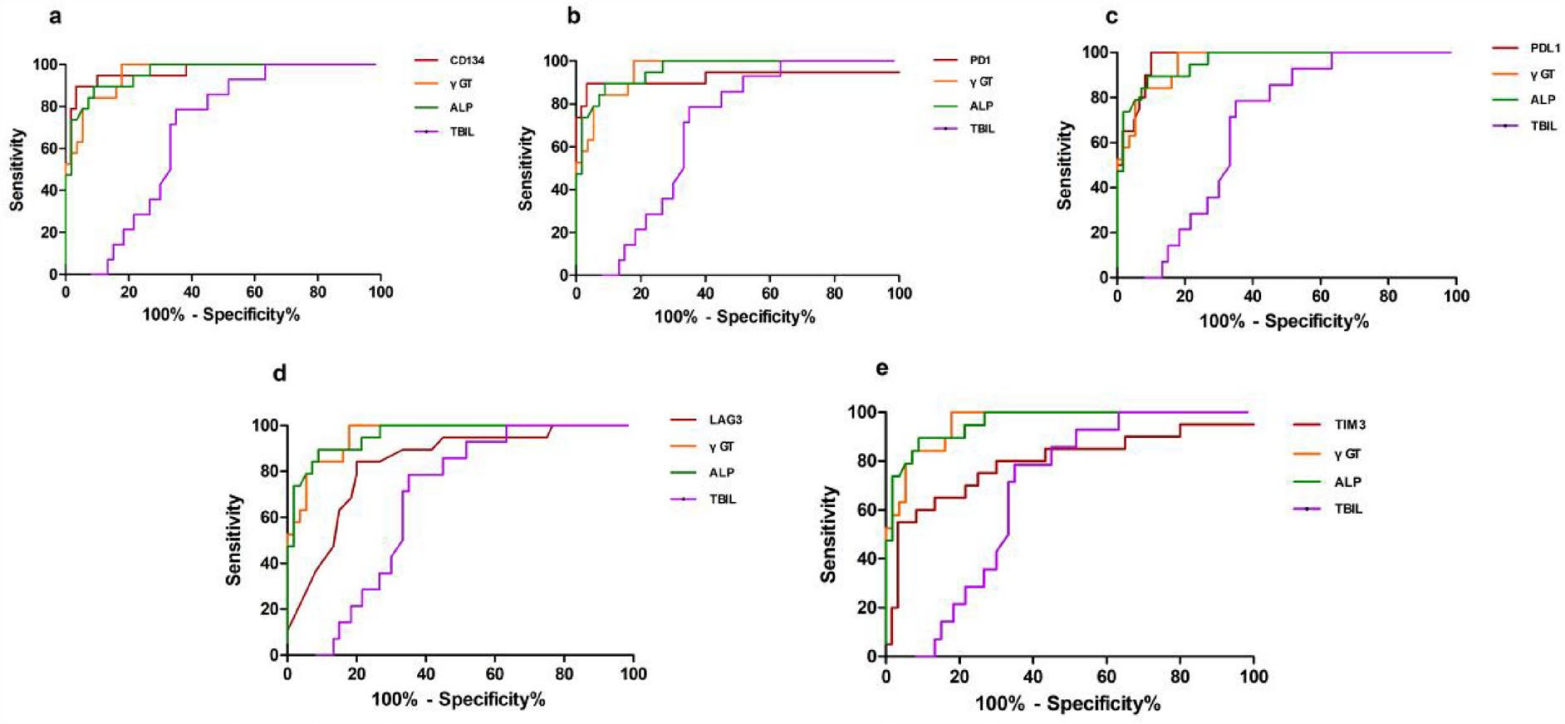
Compared with laboratory indexes, the diagnostic capabilities of the CD134 (**a**), PD-1 (**b**), PD-L1 (**c**), LAG-3 (**d**) and TIM-3 (**e**) values for PBC

**Fig. 5:**
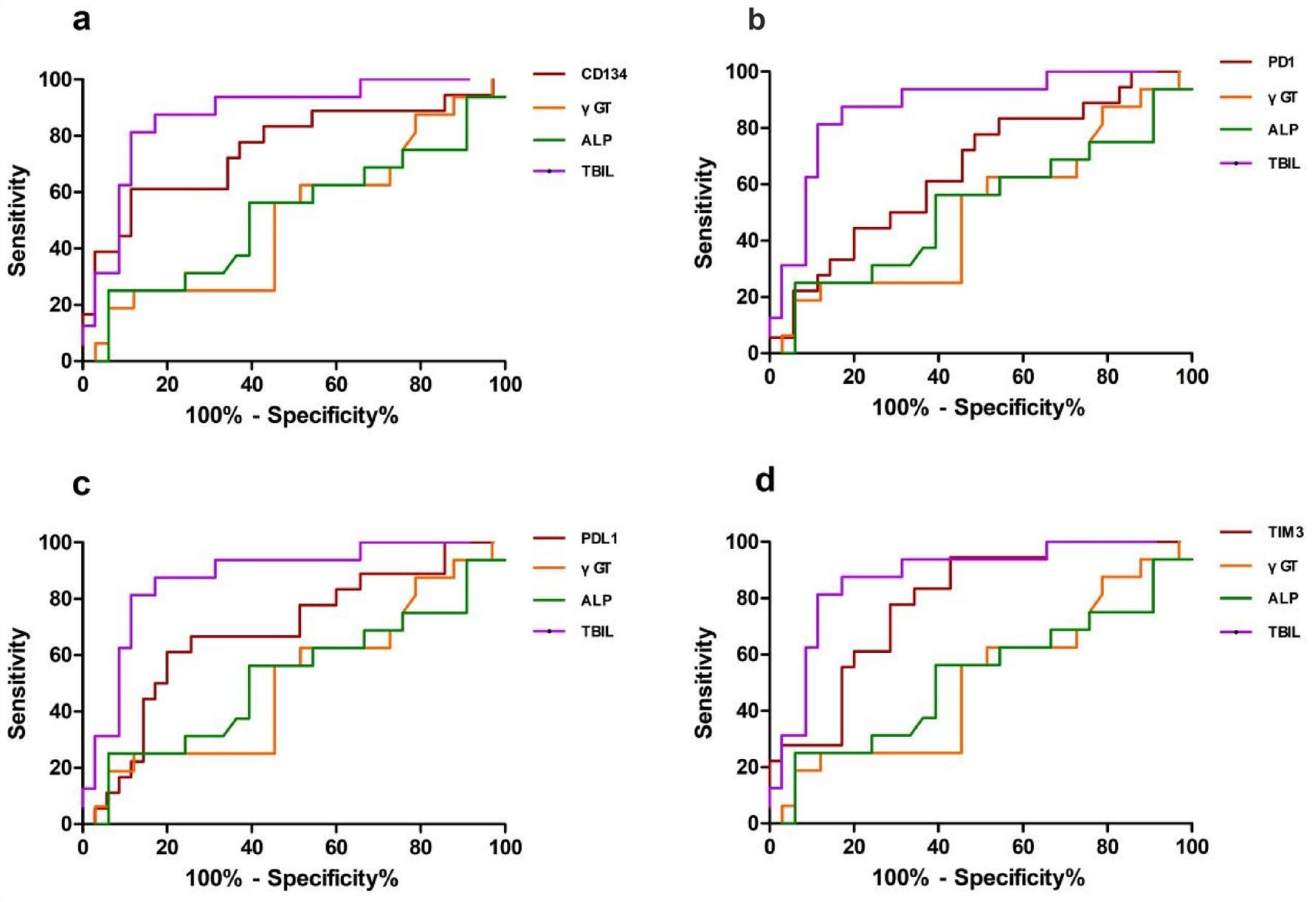
Compared with laboratory indexes, the diagnostic capabilities of the CD134 (**a**), PD-1 (**b**), PD-L1 (**c**) and TIM-3 (**d**) values for assessing the stage of PBC

Table 3 presents the calculated AUC (Area Under the Curve), optimal cut-off values, sensitivity, and specificity for PBC diagnosis and PBC severity. The AUCs for serum soluble PD-L1, OX40, PD-1, LAG-3, and TIM-3 were 0.97, 0.967, 0.922, 0.837, and 0.793, respectively. The corresponding optimal cut-off values for PBC diagnosis were 68, 89.15, 213.4, 1.05, and 252.7 pg/mL, respectively. Additionally, it provides the AUC, optimal cut-off values, sensitivity, and specificity for each soluble checkpoint in predicting the PBC stage. The AUCs for serum soluble OX40, PD-1, PD-L1, and TIM-3 were 0.767, 0.669, 0.681, and 0.765, respectively. The corresponding optimal cut-off values for predicting PBC stage ≥ III were 199.45, 302.6, 116.65, and 361.5, respectively. It was observed that the diagnostic performance of PD-L1 and CD134 was better than that of γ-GT and ALP in patients with PBC. The diagnostic performance of TIM-3 and CD134 was comparable to that of TBIL and IBIL in predicting PBC severity (Table 4). Figure 4a–e presents the ROC curves for the soluble checkpoints and other laboratory indexes for diagnosing PBC. Additionally, ROC analyses were carried out to assess the diagnostic efficiency of serum OX40, PD-1, PD-L1, and TIM-3 for predicting the stage of PBC. Figure 5a–d depicts the ROC curves for soluble OX40, PD-1, PD-L1, and TIM-3 in predicting the disease severity (stage ≥ III) of PBC.

**Table 3:** Diagnostic performance of soluble checkpoint and laboratory indexes in predicting PBC severity

		Cutoff	AUC	Sensitivity%	Specificity%
The diagnostic performance between HC and PBC	PD-L1	68	0.9704 (0.9399 to 1.001)	100	90
	CD134	89.15	0.9667 (0.9235 to 1.010)	89.47	96.67
	PD-1	213.4	0.9219 (0.8157 to 1.028)	89.47	96.67
	LAG-3	1.05	0.8373 (0.7383 to 0.9363)	84.21	80
	TIM-3	252.7	0.7925 (0.6597 to 0.9253)	65	86.67
	γGT	85	0.9577 (0.9176 to 0.9978)	100	82.14
	ALP	92.35	0.9591 (0.9172 to 1.001)	89.47	91.07
	TBIL	15.75	0.6774 (0.5570 to 0.7978)	78.57	65
The diagnostic performance between early (stage I-II) and late (stage III-IV) PBC	CD134	199.45	0.767 (0.623 to 0.911)	78.9	62.9
	TIM-3	361.5	0.765 (0.638 to 0.893)	78.9	65.7
	PD-1	302.6	0.669 (0.519 to 0.821)	78.9	51.4
	PD-L1	116.65	0.681 (0.531 to 0.832)	63.2	74.3
	LAG-3	1.35	0.594 (0.431 to 0.757)	73.68	45.71
	γGT	622	0.5028 (0.3254 to 0.6802)	25	87.88
	ALP	100	0.518 (0.3335 to 0.7024)	25	93.94
	TBIL	28.6	0.875 (0.7709 to 0.9791)	87.5	82.86

**Table 4:** Diagnostic performance of soluble checkpoints compared with laboratory indexes in predicting PBC severity

Diagnostic performance for patients with PBC		Diagnostic performance in predicting PBC severity	
	P value		*P* value
CD134 VS TBIL	> 0.05	PD-L1 VS γ-GT	< 0.01
CD134 VS IBIL	> 0.05	PD-L1 VS ALP	< 0.01
TIM-3 VS TBIL	> 0.05	CD134 VS γ-GT	< 0.05
TIM-3 VS IBIL	> 0.05	CD134 VS ALP	< 0.05

### Multivariate regression analysis of factors associated with advanced PBC

Age, ALB, and CD134, which were associated with advanced PBC in univariate analysis, were considered for inclusion in multivariable analysis. After adjusting for potential confounders, lower ALB levels (OR = 0.661, *P* = 0.003) and higher CD134 levels (OR = 1.014, *P* = 0.022) were independently associated with advanced PBC on multivariate analysis (Table 5).

**Table 5:** Multivariate regression analysis of factors associated with advanced PBC

Variable	B	S.E	Wald	P value	OR	95% CI for OR
Age	− 0.09	0.053	2.902	0.088	0.914	0.824–1.014
ALB	− 0.414	0.137	9.07	0.003	0.661	0.505–0.865
CD134	0.014	0.006	5.247	0.022	1.014	1.002–1.026

*S.E* standard error

### The expression of CD134 and TIM-3 is upregulated in the liver of patients with PBC

**Fig. 6:**
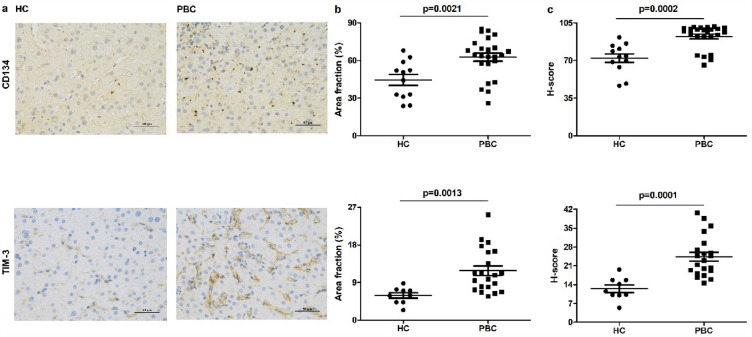
The expression of CD134 and TIM-3 is upregulated in the liver of patient with PBC. The expression of CD134 and TIM-3 in the liver of patients with PBC and healthy control (scale bar = 50 μm) **a**. Positive area ratio: positive area/tissue area (**b**), histochemistry score (**c**) of CD134 and TIM-3 in the liver of patient with PBC and healthy control

Compared with healthy controls, the expression of CD134 and TIM-3 was increased in patients with PBC, regardless of the positive area ratio or histochemistry score (Fig. 6a–c).

## Discussion

Early detection and treatment of diseases are crucial to the success of a cure. In PBC, screening patients for the status of PBC is pivotal because those with advanced disease are at greater risk of developing severe complications. Although liver biopsy is the golden standard for identifying the stage of liver fibrosis, its invasive nature limits its clinical application. The liver is a central immunological organ with a large number of myeloid and lymphoid immune cells [8]. Immune checkpoints play pivotal roles in regulating the immune response in autoimmune diseases, making them potentially useful as indicators of disease severity. In our study, we identified a series of soluble immune checkpoint-related proteins associated with laboratory indexes of patients with PBC.

CD134 (OX40, TNFRSF4) was initially identified as a T cell activation marker but was later found to be a member of the nerve growth factor receptor (NGFR) / tumour necrosis factor receptor (TNFR) superfamily with co-activation function. It is primarily expressed on activated effector T cells and regulatory T cells (Tregs), as well as natural killer T cells, natural killer (NK) cells and neutrophils. Its ligand, OX40L, is expressed on antigen-presenting cells such as dendritic and B cells. Soluble OX40 activation upregulates antigen presentation, chemokine receptor expression, and proinflammatory cytokine production by hepatic monocytes. Additionally, plasma-soluble OX40 levels are positively associated with patients with non-alcoholic steatohepatitis, revealing the clinical relevance of the findings [9]. In our study, we found that soluble CD134 was significantly increased in patients with PBC, and the titer of soluble CD134 was positively related to the severity of PBC. Moreover, we identified a positive correlation between serum soluble CD134 levels and laboratory parameters for liver injury and autoimmunity, such as IgA and IgG, TB, TBIL, and IBIL, in patients with PBC.

As another important immune checkpoint axis, PD-1/PD-L1 has achieved great success in the development of anti-tumor drugs. PD-1, also known as CD279, is a cell surface receptor belonging to the immunoglobulin superfamily and is expressed on T and B cells. PD-1 binds to its ligands, PD-L1, and functions as an immune checkpoint by inhibiting T cell function. They are considered negative immune modulators that maintain homeostasis and protect tissues from autoimmune responses. Both PD-1 and PDL have soluble forms that are produced through proteolytic cleavage of membrane-bound proteins [10]. Soluble PD-1 and PD-L1 functions were studied in malignant and non-malignant diseases [11]. Soluble programmed death-1 levels are associated with disease activity and treatment response in patients with autoimmune hepatitis in both adults and pediatric patients [12, 13]. Similarly, in our study, we found that soluble PD-1 and PD-L1 were significantly increased in patients with PBC, and the titer of soluble PD-1 and PD-L1 were positively correlated with the severity of PBC. Like soluble CD134, soluble PD-1 and PD-L1 levels positively correlate with liver function.

TIM-3, also known as HAVCR2, is an inhibitory receptor expressed on the surface membrane of activated T cells, Treg cells, macrophages/monocytes and NK cells [14, 15]. sTIM-3 is the soluble form of TIM-3 in blood circulation, derived from an alternatively spliced product of the TIM-3 gene. Soluble TIM-3 competes with membrane-expressed TIM-3 for binding to its ligand gal-9, resulting in the modulation of TIM-3-mediated immune response. However, there is limited evidence for altered sTIM-3 in PBC. Here, we hypothesized that serum sTIM-3 may reflect the severity of PBC. Consistent with our hypothesis, compared with healthy controls, the serum levels of sTIM-3 increased in patients with PBC. Correlation analysis indicated that sTIM-3 levels were not only positively associated with the stage of PBC but also positively associated with levels of TB, TBIL, IBIL, GLB and negatively associated with CHE.

LAG-3, a membrane glycoprotein, functions as a checkpoint to regulate immune cells, including T cell activation, proliferation, cytokine production, and cytolysis [16]. Numerous studies have confirmed the importance of LAG-3 in the pathogenesis of autoimmune diseases [17–19]. Mature LAG-3 can be cleaved from the cell membrane to produce another soluble form, sLAG-3, which cooperates with the activity of T cells [20]. We found that soluble LAG-3 was significantly increased in patients with PBC. Correlation analysis indicated that sLAG-3 levels were positively associated with IgG, AST, TBA and DBIL levels and negatively associated with levels of CHE.

In addition to the soluble checkpoint, we also tested the expression of checkpoint in the liver of patients with PBC and healthy control. Consistent with serological results, the expression of CD134 and TIM-3 was upregulated in the livers of patients with PBC.

To our knowledge, this study is the first to investigate the relationship between immune checkpoints and PBC staging and related biochemical indicators. These results indicate the prognostic potential of these soluble immune checkpoint-related proteins and unveiled potential biological mechanisms in PBC progression. Additionally, our study, which utilised liver biopsy samples to investigate the expression of immune checkpoints on intrahepatic lymphocytes in patients with PBC, providing a more intuitive representation of the role of immune checkpoints in the liver.

Despite these strengths, there are limitations to this study. First, the sample size is small, and the majority of patients with PBC were in the early stages (I and II), making the subanalysis in different stages underpowered. Thus, further studies with larger sample sizes are needed. Second, we did not investigate the mechanisms to determine the functional impact of soluble immune checkpoints in PBC. Lastly, the absence of positive controls, including Non-alcoholic Steatohepatitis (NASH), Chronic Hepatitis B (CHB), and viral hepatitis, also represents a limitation of this study. In forthcoming research, we plan to incorporate these patient groups as positive controls to enhance the comprehensiveness of this study.

In summary, although the mechanism of soluble immune checkpoints in PBC remains unclear, our results showed a significant increase in soluble immune checkpoints in patients with PBC compared to those in healthy controls. They were correlated with disease severity, suggesting that soluble immune checkpoints could be used as a biological indicator for measuring disease severity.

## Conclusions

Our study implies that the titer of serum soluble checkpoint may be increased in Chinese patients with PBC.

### Supplementary Information

**Additional file 1: Table S1.** Patients’ clinical characteristics and laboratory index.

## Data Availability

The datasets used and/or analysed during the current study are available from the corresponding author on reasonable request.

## Abbreviations

PBC: Primary biliary cholangitis
ELISA: Enzyme-linked immunosorbent assay
AMA: Anti-mitochondrial antibody
ROC: Receiver operating characteristic curve
HC: Healthy control
APC: Antigen-presenting cell
AST: Aspartate transaminase
ALT: Alanine aminotransferase
TBA: Total bile acids
TB: Total bilirubin
DBIL: Direct bilirubin
IBIL: Indirect bilirubin
GLB: Globulin
CHE: Cholinesterase
