# Supplementary material for: Soluble immune checkpoints are elevated in patients with primary biliary cholangitis
Source: Eur J Med Res. 2023 Nov 2;28:477. doi: 10.1186/s40001-023-01419-6 (PMC10621234; doi:10.1186/s40001-023-01419-6)
Supplement: Supplementary file 1 — Additional file 1: Table S1. Patients’ clinical characteristics and laboratory index. [file 40001_2023_1419_MOESM1_ESM.docx]

Additional file

Table S1 Patients’ clinical characteristics and laboratory index

|  |  |  | PBC | | | | | p value | p value | p value | p value | p value | p value | p value | p value | p value | p value | p value | p value |
| --- | --- | --- | --- | --- | --- | --- | --- | --- | --- | --- | --- | --- | --- | --- | --- | --- | --- | --- | --- |
|  | HC (N=20) | PBC (N=60) | 1 (N=11) | 2 (N=24) | 3 (N=8) | 4 (N=11) | unclear (N=6) | HC vs PBC | HC vs 1 | HC vs 2 | HC vs 3 | HC vs 4 | HC vs unclear | 1 vs 2 | 1 vs 3 | 1 vs 4 | 2 vs 3 | 2 vs 4 | 3 vs 4 |
| Age,mean(SE) | 53.4±1.6 | 54.4±1.2 | 56.09±3.3 | 54.17±1.9 | 48.88±3.0 | 56.91±2.7 | 54.67±2.7 |  |  |  |  |  |  |  |  |  |  |  |  |
| Sex |  |  |  |  |  |  |  |  |  |  |  |  |  |  |  |  |  |  |  |
| Male (%) | 5 (25) | 8（13.3） | 2（18.2） | 4（16.7） | 0（0） | 2（18.2） | 0（0） | 0.29 | - | - | - | - | - | - | - | - | - | - | - |
| Female (%) | 15 (75) | 52（86.7） | 9（81.8） | 20 | 8（100） | 9（81.8） | 8（100） |  |  |  |  |  |  |  |  |  |  |  |  |
| ALT（U/L, median(range)） | 22.6(9.9-109) | 55.5(13-893) | 38 (27-218) | 60 (13-893) | 120.2 (33-436) | 53 (20-103) | 43.25 (31-129) | 0.0000 | 0.0033 | 0.0001 | 0.0001 | 0.0048 | 0.0171 | 0.1802 | 0.0620 | 0.7564 | 0.2004 | 0.2328 | 0.0676 |
| γGT（U/L, median(range)） | 19.6(5-80) | 210.5(20-1218) | 295 (71-1218) | 298 (20-878) | 457.5 (95-988) | 114 (28-266) | 129.2 (74-617) | 0.0000 | 0.0000 | 0.0000 | 0.0000 | 0.0001 | 0.0000 | 0.5842 | 0.4421 | 0.0101 | 0.1835 | 0.0654 | 0.0044 |
| ALP（U/L, median(range)） | 67.2(42-128) | 186.1(67-1081) | 181 (102-646) | 238 (67-693) | 246.5 (87-1081) | 140 (78-505) | 182.1 (138.7-533) | 0.0000 | 0.0000 | 0.0000 | 0.0000 | 0.0000 | 0.0000 | 0.8452 | 0.4421 | 0.2512 | 0.5178 | 0.1864 | 0.1220 |
| AST（U/L, median(range)） | 24(17.1-66) | 76(21-730) | 60 (22-145) | 71 (21-282) | 93.7 (37-730) | 70.5 (39-165) | 59.1 (36-88.1) | 0.0000 | 0.0002 | 0.0000 | 0.0000 | 0.0000 | 0.0002 | 0.9838 | 0.3950 | 0.5116 | 0.1500 | 0.5588 | 0.4082 |
| Glb（g/L, median(range)） | 29.3(25.3-42) | 35(23-64) | 34 (27-44) | 34.2 (28-42) | 37.6 (30-64) | 36.5 (23-43) | 31.95 (25.7-44.8) | 0.0004 | 0.0064 | 0.0008 | 0.0025 | 0.0623 | 0.0948 | 0.7995 | 0.1518 | 0.9725 | 0.0390 | 0.6357 | 0.3154 |
| TB（μmol/L, median(range)） | 14.05(7.7-32.6) | 27.6(5.5-282.8) | 14.3 (9.3-85.4) | 16.4 (5.5-77.7) | 69.95 (20-157.8) | 66.1 (29-282.8) | 28.7 (12-145.2) | 0.0035 | 0.3445 | 0.3587 | 0.0000 | 0.0000 | 0.0200 | 0.7606 | 0.0018 | 0.0006 | 0.0004 | 0.0001 | 0.6965 |
| Dbil（μmol/L, median(range)） | 2.35(1.4-4.9) | 11.2(2-168.3) | 5.2 (3.1-53.8) | 5.65 (2-52.6) | 44.3 (4.8-106) | 30.6 (10-168.3) | 12.05 (4.4-102.3) | 0.0000 | 0.0000 | 0.0001 | 0.0000 | 0.0000 | 0.0002 | 0.8232 | 0.0091 | 0.0011 | 0.0057 | 0.0006 | 0.8286 |
| IBil（μmol/L, median(range)） | 11.85(6.3-27.7) | 13(3-114.5) | 10.3 (5.2-31.6) | 9.2 (3-29) | 22.8 (6.1-51.8) | 30.8 (16.5-114.5) | 15.6 (7.6-42.9) | 0.4161 | 0.6475 | 0.1482 | 0.0502 | 0.0000 | 0.3530 | 0.3375 | 0.0506 | 0.0008 | 0.0081 | 0.0000 | 0.5148 |
| TBA（μmol/L, median(range)） | 2.85(1.7-14.8) | 34(0.4-268.6) | 12.5 (2.3-108) | 17.3 (0.4-8.7) | 139.8 (10.5-268.6) | 68.5 (14.2-255) | 37.2 (3.1-51.6) | 0.0000 | 0.0028 | 0.0010 | 0.0000 | 0.0000 | 0.0033 | 0.7670 | 0.0203 | 0.0101 | 0.0185 | 0.0034 | 0.4082 |
| CHE（U/L, median(range)） | 8495(6729-12532) | 5945(374-12580) | 8443 (4789-12580) | 7063 (374-11988) | 4307 (1964-6849) | 2342 (1505-3782) | 5333 (2060-9660) | 0.0006 | 0.5357 | 0.0273 | 0.0000 | 0.0000 | 0.0117 | 0.2608 | 0.0005 | 0.0000 | 0.0057 | 0.0000 | 0.0117 |
| IgA（g/L, median(range)） | NA | 3.2(1.4-9.6) | 3 (1.9-5.1) | 2.95 (2-5.6) | 3.2 (2.3-5.5) | 5.4 (1.4-9.6) | 4 (2.5-6) | - | - | - | - | - | - | 0.9623 | 0.7104 | 0.0530 | 0.6009 | 0.0553 | 0.0728 |
| IgG（g/L, median(range)） | NA | 15.9(10.5-56.1) | 16.2 (14.4-23.2) | 14.65 (12.5-24.6) | 17.9 (14.3-56.1) | 15.6 (11.9-23.9) | 19.9 (10.5-23.2) | - | - | - | - | - | - | 0.2295 | 0.3829 | 0.9015 | 0.0330 | 0.4747 | 0.3176 |
| IgM（g/L, median(range)） | NA | 2.7(0.8-8.1) | 2.5 (1.9-4.3) | 3.75 (0.8-8.1) | 5 (2.1-6.7) | 2.7 (1.1-4.7) | 2.4 (2.2-2.7) | - | - | - | - | - | - | 0.5362 | 0.1649 | 0.9015 | 0.8868 | 0.3638 | 0.1282 |
